# Impairment of vascular strain in patients with obstructive sleep apnea

**DOI:** 10.1371/journal.pone.0193397

**Published:** 2018-02-28

**Authors:** Max Jonathan Stumpf, Christian Alexander Schaefer, Jan Krycki, Robert Schueler, Carmen Pizarro, Georg Nickenig, Martin Steinmetz, Dirk Skowasch, Izabela Tuleta

**Affiliations:** Department of Internal Medicine II–Cardiology, Pulmonology and Angiology, University of Bonn, Bonn, Germany; National Yang-Ming University, TAIWAN

## Abstract

**Background:**

Obstructive sleep apnea (OSA) is an independent risk factor for the development of cardiovascular diseases. Aim of this present study was to evaluate and extend recent research on the influence of obstructive sleep apnea on vascular strain.

**Methods:**

A total number of 98 patients were integrated in the study. Patients were grouped according to the Apnea-Hypopnea-Index (AHI) in patients with mild-to-moderate OSA (5/h ≤ AHI < 30/h), severe OSA (AHI ≥ 30/h) and controls (AHI < 5/h). Groups were matched in age, body-mass-index and cardiovascular risks. Vascular strain of common carotid arteries was assessed by ultrasound speckle-tracking. A minor group of 30 patients and controls further underwent assessment of vascular strain of brachial and femoral arteries. Additionally, all patients underwent blood testing to reveal potential influences of inflammatory markers on arterial stiffness. In additional analysis we examined the effect of statin therapy on vascular strain.

**Results:**

Patients with OSA showed significantly reduced values of vascular strain of common carotid arteries. Radial and circumferential strains were significantly lower in both patients with mild-to-moderate (p = .05) and patients with severe OSA (p = .001) compared to control. Vascular strain parameters of brachial and femoral arteries showed no consistent results. There were no significant correlations of inflammatory markers with vascular strain parameters. No significant differences in vascular strain were detected between statin and non-statin groups.

**Conclusion:**

Patients with OSA show significantly reduced vascular strain assessed by ultrasound-based speckle-tracking. Vascular stiffness increases with the severity of the disease. Target vessels to assess vascular strain in patients with OSA are common carotid arteries, whereas other sites of the arterial tree are not reliable. No significant impact of current statin therapy on vascular strain was found. Further studies are needed to evaluate potential benefit of statins in secondary prevention of atherosclerosis in OSA.

## 1. Introduction

Obstructive sleep apnea (OSA) is a widespread chronic disease which affects up to 17% of the population in industrialized countries [1, 2]. It is independently correlated with metabolic dysregulations such as diabetes, arterial hypertension, hypercholesterolemia and obesity, as well as increased risk for cardiovascular events and atherosclerosis [3–5]. Also, a connection to Alzheimer's disease is frequently reported [6, 7]. OSA is characterized by repetitive total or partial collapse of upper airways during sleep with consecutive phases of oxygen deficiency and resaturation. Intermittent hypoxia (IH) and resaturation generate oxidative stress leading to endothelial tissue damages, endothelial dysfunction and various inflammatory processes [8–10]. Severe consequences of OSA are coronary artery disease (CAD) and peripheral arterial occlusive disease (pAOD) which have been described multiple times in previous research [3, 5, 11, 12]. A sensitive noninvasive method to measure stiffness of the arterial wall is the analysis of vascular strain, based on ultrasound-based speckle-tracking [13–16]. In a recent pilot study, Tuleta et al. [17] were able to show a relation between vascular strain of common carotid arteries (CCA) and OSA. The aim of this present study was to confirm and extend these previous results in a greater, and thus, more representative OSA collective. Beyond this, the value of vascular strain analysis performed at other sites of the arterial tree was evaluated. Further, potential influence of inflammation and statin therapy of vascular strain was examined.

## 2. Methods

This study was conducted according to the principles of the Declaration of Helsinki for Human Research and was approved by the local ethics committee of the University Hospital of Bonn. Written informed consent of all patients and controls was obtained prior to the examination. The Department of Pneumology and the Department of Angiology of the University Hospital of Bonn realized this study as a joint project.

### 2.1 Patients and controls

From November 2016 till March 2017 126 consecutive patients with suspected OSA were examined. The diagnosis was confirmed via polysomnography. Thereby, apnea was defined as an air flow cessation for at least 10 seconds and hypopnea as an air flow reduction of minimum 50% for 10 seconds or more accompanied by an oxygen desaturation of > 3% or a cortical arousal which matches the definition of hypopnea of the American Academy of Sleep Medicine from 2012 [18,19]. Classification of patients with diagnosed OSA was performed according to the Apnea-Hypopnea-Index (AHI) which derives from the sum of apnea and hypopnea events per hour of sleep. Exclusion criteria were central sleep apnea (n = 20) and known OSA with current CPAP treatment (n = 6). Two patients had to be excluded due to massive thyroid struma (n = 1) and increased shortage of breath (n = 1). In both cases no analyzable vascular strain could be assessed. Accordingly, 98 patients were included in the study. Patients under current therapy with statins (n = 27) were separated from the main group and were part of additional analysis. 28 patients with an AHI ≥5/h and <30/h formed a group of mild-to-moderate OSA. Patients with an AHI ≥30/h were diagnosed with severe OSA (n = 25). 18 patients with an AHI <5/h measured by polysomnography served as a control group (control). All participating patients were naive to treatment (e.g. CPAP-therapy).

### 2.2 Polysomnography

All patients underwent cardiorespiratory polysomnography (Somnolab, Weinmann Medical Technology, Hamburg, Germany). Baseline examination included pulse oxymetry, electrophysiological surveillance (EEG, EOG, EMG) and ECG. Surveillance of sleep contained the recording of sleep phases and sleep stages, snoring [%/time of sleep], awakening reactions expressed as the total arousal index (TAI) [n/h], thoracic movement observation and the periodic limb movement during sleep (PLMS) [n/h]. AHI, which is described above, and Oxygen-Desaturation-Index (ODI) were of particular interest for this study. ODI expresses the oxygen desaturation phases (decrease of oxygen saturation > 3%) per hour.

### 2.3 Standard angiological examinations

Angiological diagnostics contained evaluation of Ankle-Brachial-Index (ABI) and color-coded duplex sonography of the arteries. ABI was defined as quotient of systolic blood pressure of tibial posterior artery and dorsalis pedis artery on both sides through mean systolic blood pressure of the upper limbs. An ABI value <0.9 was considered an indication for peripheral artery occlusive disease (pAOD), ABI >1.3 was interpreted as a lead to increased vascular stiffness. Color-coded duplex sonography was performed on carotid arteries, brachial arteries, aorta and the arteries of the lower limb down to popliteal artery using Philips® iE33® (Hamburg, Germany). Presence of one or more atherosclerotic lesions (plaques) in carotid arteries led to the diagnosis of cervical arterial occlusive disease (cAOD), one or more plaques in the arteries of the lower limb indicated peripheral artery occlusive disease (pAOD). Sonography was performed by skilled physicians who were blinded to the results of polysomnography.

### 2.4 Vascular strain analysis

Vascular strain analysis was performed on CCA one centimeter below the carotid bulb. Two-dimensional cross-sectional grey-scaled recordings of at least four consecutive heart beats were collected, triggered by electrocardiography (ECG). Assuming bilateral equal alterations of arteries as consequence of OSA the clip of the highest quality was chosen for further evaluation. Thereby, clips were transferred as DICOM file to an external workstation equipped with the speckle-tracking software package Image Arena®, Version 4.6 by TomTec Systems GmbH (Munich, Germany) for off-line analysis.

For measurement of circumferential and radial strain variables a region of interest (ROI) was marked manually in the intima-media complex of the vessel wall, adequate tracking was verified and adjusted, if necessary [13, 15, 20]. Afterwards, the vessel wall was divided in six equally sized parts, whereupon strain parameters where collected of each part. We considered the following averaged parameters for further analysis: radial displacement (r.Dis) as a description of overall wall movement [mm], radial strain (r.Str) representing radial expansion of the vessel wall during the cardiac cycle [%], circumferential strain (c.Str) characterizing the change of the vessel wall circumference during one heart cycle [%], radial velocity (r.Vel) [cm/s], radial strain rate (r.StrR) [1/s] and circumferential strain rate (c.StrR) [1/s] as dynamic parameters of the arterial wall motion over time. Displacement and strains resulted from the sum of minimum and maximum average values of each of those parameters. Radial velocity and strain rates were assessed as maximum change rates [20].

Within the main group of patients and controls without current statin therapy a total number of 30 consecutive patients underwent extended vascular strain analysis. Of those, 12 patients presented with mild-to-moderate OSA, 11 patients suffered from severe OSA, and 7 patients were diagnosed free of OSA. This analysis aimed to evaluate the value of brachial and femoral arteries in assessment of vascular strain in patients with OSA. For this purpose vascular strain was assessed not only in common carotid arteries, but additionally in the brachial arteries at the axial upper arm as well as in the femoral arteries one centimeter distal to the bifurcation of common femoral arteries.

### 2.5 Blood testing

Differential blood count, total cholesterol, high density lipoprotein (HDL), low density lipoprotein (LDL), high sensitive C-reactive protein (hsCRP), lipoprotein a (Lip(a)), interleukin-6 (IL-6), fibrinogen and soluble interleukin-2-receptor (sIL-2r) were determined in blood. All parameters have previously been described in relation to increased arterial thickness respectively atherosclerosis in patients with OSA [21–25].

### 2.6 Influence of statins on vascular strain

27 patients with current statin therapy were separated from the main group and integrated in the subgroup analysis. Of those, patients with an AHI < 5 were not included in further evaluation (n = 7). As control group served a sample of 20 patients gathered by chance with SPSS® out of all study participants (main group) with diagnosed OSA and without current statin therapy. The groups showed neither significant differences in BMI, packyears, age nor AHI and ODI. By comparing these groups we intended to glance at possible influences of statin therapy on vascular stiffness in patients with OSA and therefore generate new hypotheses for future investigations.

### 2.7 Statistical analysis

Statistical analysis was conducted with IBM® SPSS® Statistics®, Version 23. Intergroup differences of nominal scaled baseline characteristics were calculated by Chi-square-test and Cramer's V. Comparing the groups on behalf of Gaussian-distributed parameters was performed by ANOVA, non Gaussian-distributed parameters under went Mann-Whitney-U- and Kruskal-Wallis-tests. Correlation between vascular strain and blood parameters, respectively partial correlation related to PLMS, was performed by Spearman's rho. Two-tailed p-value was defined significant at the .05-level. Continuous variables were presented as mean ± standard deviation.

Dissenting from that, subgroup analysis between patients with or without current statin therapy was performed by t-test for uncombined samples instead of ANOVA on behalf of Gaussian distributed parameters.

## 3. Results

Baseline characteristics are presented in Table 1. There were no significant differences in age, body mass index (BMI) and cardiovascular risk factors. The group of patients with severe OSA comprised significantly more men than the control group which mirrors the male sex as a risk factor for OSA. Although without significance, the prevalence of atherosclerotic lesions was clearly elevated in patients with OSA.

**Table 1:** Baseline characteristics.

	Mild-to-moderate OSA (n = 28)		Severe OSA (n = 25)		Control (n = 18)
	Value	p*	Value	p*	Value
Sex: male [%]	20 [71,4]	n. s.	22 [88,0]	< .05	9 [50,0]
Age [Years]	58.2 ± 11.1	n. s.	54.5 ± 14.2	n. s.	51.7 ± 14.0
BMI [kg/m^2^]	30.98 ± 5.72	n. s.	32.67 ± 5.55	n. s.	28.98 ± 4.77
AHI [n/h]	14.1 ± 6.7	< .001	55.9 ± 19.2	< .001	2.1 ± 1.7
ODI [n/h]	14.2 ± 7.4	< .001	52.4 ± 22.3	< .001	2.2 ± 2.2
PLMS [n/h]	17.7 ± 27.6	n. s.	12.0 ± 17.5	n. s.	13.1 ± 30.9
TAI [n/h]	23.4 ± 13.1	n. s.	38.6 ± 27.1	< .001	12.4 ± 21.4
Snoring [%/time of sleep]	34.1 ± 32.3	< .01	28.7 ± 23.4	< .05	8.7 ± 18.3
Nicotine abuse** [%]	20 [71.4]	n. s.	13 [52.0]	n. s.	6 [33.3]
Packyears [Years]	18.8 ± 21.8	< .01	11.6 ± 21.3	n. s.	4.9 ± 10.0
Diabetes [%]	2 [7.1]	n. s.	4 [16.0]	n. s.	1 [5.6]
Hypertension [%]	21 [61.1]	n. s.	17 [68.0]	n. s.	11 [61.1]
Hypercholesterolemia [%]	8 [28.6]	n. s.	3 [12.0]	n. s.	3 [16.7]
Renal dysfunction [%]	0		0		0
CAD [%]	2 [7.1]	n. s.	2 [8.0]	n. s.	0
Myocardial infarction [%]	2 [7.1]	n. s.	1 [4.0]	n. s.	0
cAOD [%]	15 [53.6]	n. s.	12 [48.0]	n. s.	5 [27.8]
pAOD [%]	16 [57.1]	n. s.	10 [40.0]	n. s.	7 [38.9]
ABI < .90 [%]	4 [14.3]	n. s.	5 [20.0]	n. s.	1 [5.6]
ABI > 1.30 [%]	6 [21.4]	n. s.	4 [16.0]	n. s.	5 [27.8]

* vs. control

** current and former nicotine abuse

*BMI* Body-Mass-Index; *AHI* Apnea-Hypopnea-Index; *ODI* Oxygen-Desaturation-Index; *PMLS* Periodic limb movement during sleep; *TAI* Total arousal index; *CAD* coronary artery disease; *cAOD* cervical arterial occlusive disease; *pAOD* peripheral arterial occlusive disease; *ABI* ankle-brachial-index

### 3.1 Vascular strain analysis

**Fig 1:**
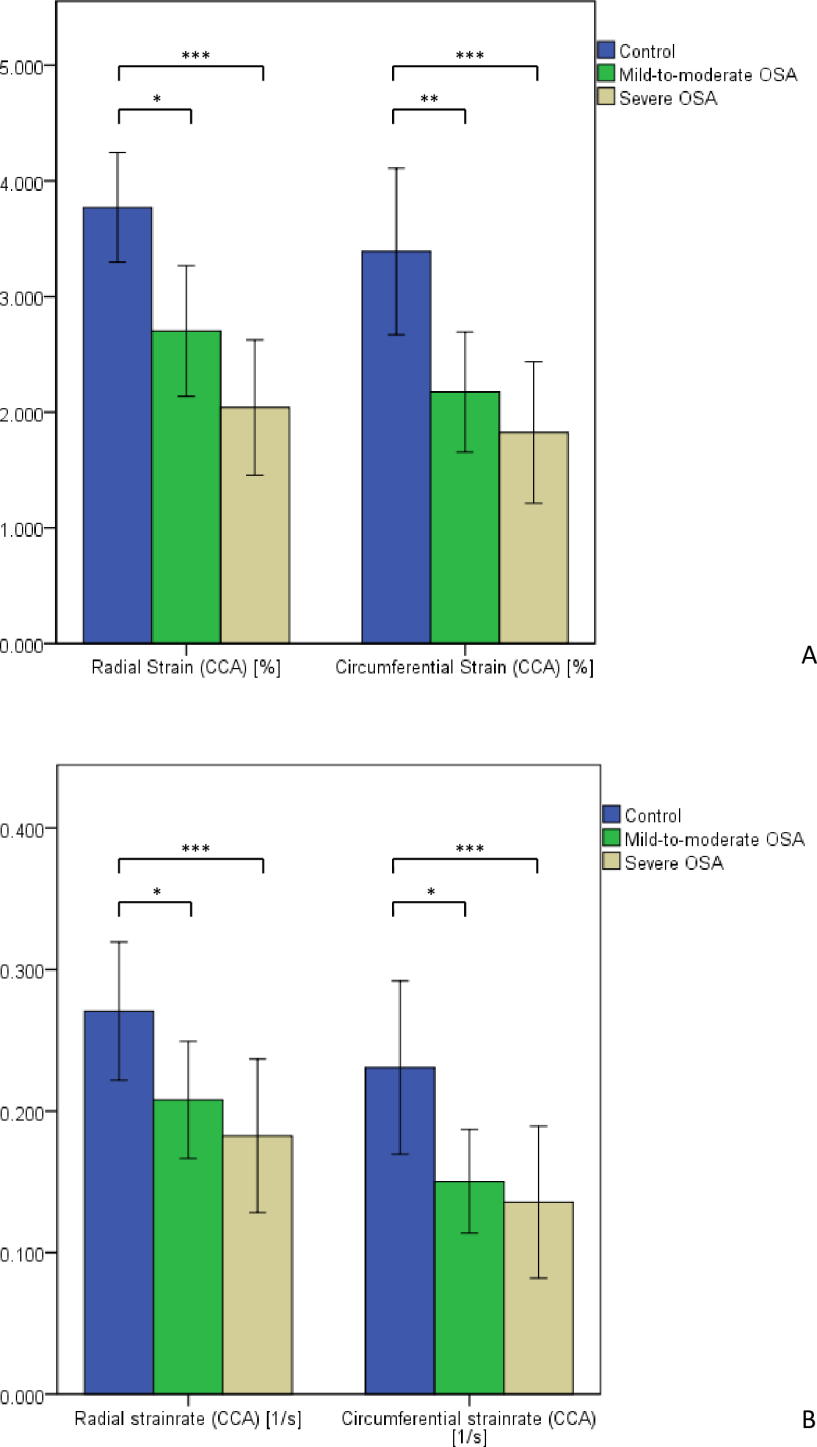
**Results of vascular strain analysis of common carotid arteries (CCA):** *A* radial and circumferential strain; *B* radial and circumferential strain rate; * p < .05; ** p < .01; *** p < .001.

Results of vascular strain analysis of common carotid arteries are illustrated in Table 2 and Fig 1. Radial velocity was significantly lower values in patients with mild-to-moderate OSA compared to controls (p < .05) and decreased in patients with severe OSA (p < .01). Radial displacement presented lower in mild-to-moderate OSA yet without significance. In patients with severe OSA r.Dis decreased significantly (p < .01). Radial strain and circumferential strain were significantly reduced in both mild- to-moderate and severe OSA compared to control and reached a highly significant level in patients with severe OSA (p < .001). This counted for circumferential strain rate as well.

**Table 2:** Results of vascular strain analysis of common carotid arteries.

	Mild-to-moderate OSA		Severe OSA		Control
	Value	p*	Value	p*	Value
r.Vel** [cm/s]	.070 ± .049	< .05	.059 ± .054	< .01	.094 ± .054
r.Dis [mm]	.100 ± .065	n. s.	.076 ± .060	< .01	.136 ± .061
r.Str [%]	2.702 ± 1.457	< .05	2.041 ± 1.414	< .001	3.771 ± .952
c.Str** [%]	2.175 ± 1.338	< .01	1.825 ± 1.485	< .001	3.390 ± 1.448
r.StrR [1/s]	.208 ± .107	n. s.	.182 ± .131	< .05	.271 ± .098
c.StrR** [1/s]	.150 ± .094	< .01	.136 ± .130	< .001	.231 ± .123

* vs. control

** non-Gaussian distributed

*n*. *s*. not significant; *r*.*Vel* radial velocity; *r*.*Dis* radial displacement; *r*.*Str* radial strain; *c*.*Str* circumferential strain; *r*.*StrR* radial strain rate; *c*.*StrR* circmferential strain rate

**Fig 2:**
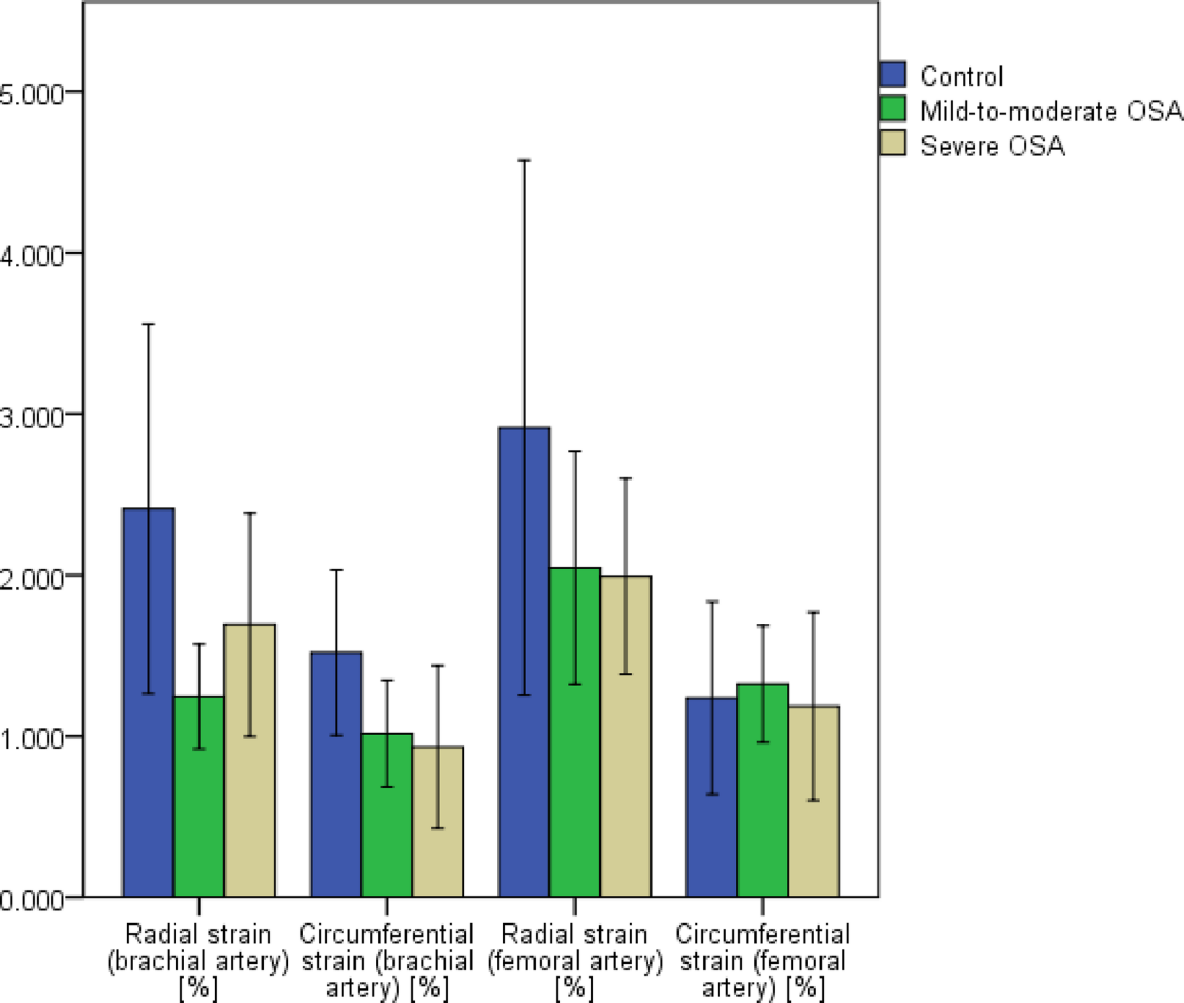
Results of vascular strain analysis of brachial and femoral arteries: radial and circumferential strain.

Results of vascular strain analysis at different sites of the arterial tree are presented in S1 Table and Fig 2. No significant differences in age, BMI and packyears occurred between the groups. Vascular strain analysis of common carotid arteries counterfeited the results of the main group analysis. r.Dis, r.Str and c.Str reached a p-value of p < .001 comparing severe OSA with control. Results of vascular strain analysis of brachial arteries and femoral arteries in patients with mild-to-moderate and severe OSA were inconsistent and showed no significant differences in comparison to controls. Moreover, single parameters did not display the severity of OSA.

### 3.2. Blood testing

Blood testing is shown in S2 Table. Patients with severe OSA showed marginal higher values of total cholesterol and LDL. CRP was elevated in patients with OSA, but without statistical relevance. Monocytes presented significantly increased in patients with severe OSA (p < .01). Hematocrit (Hkt) was significantly higher at the .05-level. Lip(a), IL-6, sIL-2r, and fibrinogen showed no intergroup discrepancies at any level of significance.

### 3.3 Correlation between AHI, ODI, PLMS, TAI, and snoring with vascular strain and blood parameters

AHI correlated positively with BMI (< .01), total cholesterol (p < .05) and monocytes (p < .001). AHI was related negatively to radial velocity (p < .05), radial displacement (p <. 01), radial strain (p < .001), circumferential strain (p < .01), radial strain rate (p < .05) and circumferential strain rate (p < .01). Similarly, ODI correlated positively with BMI (p < .01), CRP (p < .05) and monocytes (p < .001). Negative correlations occurred with r.Vel (p < .05), r.Dis (p < .01), r.Str (p < .001), c.Str (p < .01) as well as radial and circumferential strain rates (p < .05).

PLMS correlated with neither vascular strain nor blood parameters at any level of significance. Monocyte count correlated with TAI (p < .01) and snoring (p < .05). However, this was expected, since TAI and AHI correlated positively with the highest significance (p < .001). To clarify, if PLMS had an influence on correlation between vascular strain and blood parameters, partial correlation with PLMS as controlled variable was calculated. As a result, correlation between vascular strain and blood parameters remained still unchanged.

There were no significant correlations between derivates of vascular strain and blood parameters.

### 3.4 Influence of statins on vascular strain

Additional analysis of patients with current statin therapy versus patients without current statin therapy is merged in S3 Table. There were no significant differences in vascular strain parameters between statin and non-statin group. However, all derivates of vascular strain presented a trend towards higher values in patients with current statin therapy. Blood testing revealed expected differences in total cholesterol [mg/dl] (statin-group: 171 ± 30 vs. non-statin group: 210 ± 36; p < .001) and LDL [mg/dl] (statin group: 107 ± 31 vs. non-statin group: 139 ± 34; p < .01). No differences in HDL, CRP and other assessed blood parameters occurred.

## 4. Discussion

OSA affects arterial stiffness [3, 26] assessed by vascular strain analysis [17]. This present study was able to reproduce these previous findings. All assessed strain parameters decreased significantly in patients with OSA and mirrored the severity of the disease. This study shows significantly reduced values of vascular strain not only in patients with severe OSA, but also in patients with mild-to-moderate OSA compared to controls. These results implicate that arterial stiffening expressed as decreasing values of vascular strain in patients with OSA accelerates with the severity of the disease.

Vascular strain analysis at different sites of the arterial tree has been described before by Charvat-Resl et al. [14]. Herein, the feasibility of vascular strain measurements in differently bedded arterial vessels in healthy young adults has been shown. In this present study, only vascular strain analysis of CCA provided plausible results. In contrast, vascular strain analyses of brachial and femoral arteries were inconsistent and did not mirror the severity of OSA. Since merely scarce data exist on the assessment of vascular strain in peripheral arteries, there is no standardized method to assess it. Moreover, the determination of vascular strain parameters in peripheral arteries in patients with OSA is technically difficult due to high BMI [27, 28]. Furthermore, the precise identification and designation of the ROI is limited, because the discrimination between intima, media and adventitia of the vessel walls is not easy in less superficial and smaller arteries. Additionally, the development of carotid atherosclerosis in connection with OSA is independently associated with heavy snoring. Snoring-induced vibratory trauma causes endothelial dysfunction and is specific for the throat-near lying vessels e.g. CCA, but not for peripheral arteries such as femoral arteries [29, 30]. In conclusion, vessels of outstanding interest in assessing vascular strain are common carotid arteries. Brachial and femoral arteries do not allow a reliable discrimination either within the severity of OSA or compared to control and therefore, should not be recommended for the ultrasound-based speckle-tracking analysis of vascular strain in patients with OSA.

The role of inflammatory markers in OSA has been the subject of various other experimental and clinical studies [10, 22, 25, 26]. In this present study, CRP tended to be elevated in patients with severe OSA and also correlated significantly with ODI. IL-6, another commonly reported parameter in OSA, presented with neither significant correlation nor elevated values in OSA compared with controls. The role of IL-6 in initiation and progression of OSA remains unclear in the literature [31, 32]. The influence of OSA on lipid profile and its impact on the development of atherosclerosis remains a matter of discussion up to now [33]. However, most evidence suggests that OSA and dyslipidemia are closely connected [12, 34]. Present data support this hypothesis. In this collective total cholesterol and LDL increased in patients with OSA. An elevation of erythrocytes and Hkt in patients with OSA has been reported before [35]. This study underlines these previous findings, though with a lesser extent, because the values of erythrocytes and Hkt in mild-to-moderate and severe OSA as well as control gathered within the normal range. Yet, increased hematocrit level and therefore increased blood viscosity may play a role in the development of cardiovascular events.

In differential blood count only monocytes were significantly elevated in patients with severe OSA. This may connect to Alvarez-Martins et al. [36] who observed an increased number of multipotent hematopoietic progenitor cells in a rat model of OSA. They further reported that intermittent hypoxia induces vascular remodeling in the bone marrow microenvironment by stimulating an increase of erythropoiesis and circulating monocytes. However, this present study did not manage to show a correlation between vascular strain and inflammatory blood parameters, whereas AHI and ODI correlated highly significantly with vascular strain parameters. This leads to the conclusion that impairment of vascular strain is effected either directly by chronic intermittent hypoxia or by mediators not mirrored by this study [37].

Drakatos et al. reported in 2016 that patients with OSA and increased periodic leg movement during sleep had significantly increased values of arterial stiffness assessed by photoplethysmography [38]. Present data does not support these finding. PLMS neither showed a correlation with vascular strain or blood parameters nor mirrored the severity of OSA. Additionally, there was no influence of PLMS on the correlation between vascular strain and inflammatory blood parameters.

Comparing patients currently undergoing statin therapy with a matched sample of patients without statins stimulated further thought. All assessed vascular strain parameters were higher in the statin group compared to the non-statin one. However, no level of significance has been reached. Expectedly, it became evident that statins reduced the levels of total cholesterol and LDL. An effect on other blood parameters could not be observed. However, due to the small sample size, the informative value of this analysis can be seen as limited. Moreover, there was no discrimination between doses, duration of statin treatment and agent. Reports on the value of statins in secondary prevention of atherosclerosis in patients with OSA are rare and inconsistent [39, 40]. An experimental double-blinded panel study on patients with OSA over at least one year to evaluate the influence of statins on vascular strain in patients with OSA is suggested.

Referring to Buratti et al. 2014 and 2016, who reported on an association between OSA and Alzheimer's disease [6, 7], this present study provides further information on this issue. Although without statistical significance compared to controls, around 50% of patients with OSA suffered from cAOD. This matches the results of Wendell et al. 2012, who described carotid atherosclerosis as a risk factor for dementia and Alzheimer's disease [41].

This study has several limitations. The preeminent limitation, especially with regard to the subgroups, is the samples size. Also BMI increased with the severity of OSA. Although the differences did not reach a level of significance, this may have influenced the results, since BMI correlated with vascular strain parameters and represents an independent risk factor for arterial stiffening and atherosclerosis. This refers to smoking habits as well, for patients with mild-to-moderate OSA presented with significantly more packyears compared to controls. Another limitation is the definition of hypopnea being a matter of discussion up to now [18, 19].

In conclusion, obstructive sleep apnea is a highly complicated disease for which the consequences are even more complex than the causes. To our knowledge this study is the largest clinical evaluation of vascular strain in patients with OSA up to now. The outstanding role of common carotid arteries in assessing of vascular strain has been highlighted. Other sites of the arterial tree have been shown to be unreliable and their value has been discussed critically. Further, this study was able to provide new information on the role of statin therapy in patients with OSA. Moreover, this study contributed to the implementing of vascular strain analysis into clinical routine.

Concluding, with regard to the high prevalence of pAOD and cAOD in this patient collective, it has to be underlined, how important a routine angiological screening of patients with OSA might be.

## Supporting information

S1 Table Results of vascular strain analysis at different sites of the arterial tree. * vs. control; ** non-Gaussian distributed; *BMI* Body-Mass-Index; *AHI* Apnea-Hypopnea-Index; *ODI* Oxygen-Desaturation-Index; *r*.*Vel* radial velocity; *r*.*Dis* radial displacement; *r*.*Str* radial strain; *c*.*Str* circumferential strain; *r*.*StrR* radial strain rate; *c*.*StrR* circumferential strain rate.
(DOCX)

S2 Table Results of blood testing. * vs. control; ** non-Gaussian distributed; *HDL* high density lipoprotein; *LDL* low density lipoprotein; *Lip(a)* lipoprotein a; *CRP* high sensitive C-reactive protein; *IL-6* interleukin-6; *IL-2-r* interleukin-2-receptor; *Fib* fibrinogen; Leu leucocytes; *Ery* erythrocytes; *Hb* hemoglobin; *Hkt* hematocrit; *Thrombo* thrombocytes; *Neutro* neutrophiles; *Lymph* lymphocytes; *Mono* monocytes; *Eos* eosinophiles; *Baso* basophiles.
(DOCX)

S3 Table Subgroup analysis statin vs. non-statin. * non-Gaussian distributed; *n*. *s*. not significant; *BMI* Body Mass Index; *AHI* Apnea-Hypopnea-Index; *ODI* Oxygen-Desaturation-Index; *HDL* high density lipoprotein; *LDL* low density lipoprotein; *CRP* high sensitive C-reactive protein; *Mono* monocytes; *Eos* eosinophiles; *Baso* basophiles; *r*.*Vel* radial velocity; *r*.*Dis* radial displacement; *r*.*Str* radial strain; *c*.*Str* circumferential strain; *r*.*StrR* radial strain rate; *c*.*StrR* circumferential strain rate.
(DOCX)
