# Supplementary material for: Impairment of vascular strain in patients with obstructive sleep apnea
Source: PLoS One. 2018 Feb 28;13(2):e0193397. doi: 10.1371/journal.pone.0193397 (PMC5831412; doi:10.1371/journal.pone.0193397)
Supplement: S1 Table — * vs. control; ** non-Gaussian distributed; BMI Body-Mass-Index; AHI Apnea-Hypopnea-Index; ODI Oxygen-Desaturation-Index; r.Vel radial velocity; r.Dis radial displacement; r.Str radial strain; c.Str circumferential strain; r.StrR radial strain rate; c.StrR circumferential strain rate. (DOCX) [file pone.0193397.s001.docx]

|  | Mild-to-moderate OSA (n = 12) | | Severe OSA (n = 11) | | Control (n = 7) |
| --- | --- | --- | --- | --- | --- |
|  | **Value** | **p*** | **Value** | **p*** | **Value** |
| Age [years] | 59 ± 11 | n. s. | 55 ± 16 | n. s. | 51 ± 15 |
| BMI [kg/m^2^] | 29.7 ± 6.7 | n. s. | 30.5 ± 5.3 | n. s. | 30.6 ± 6.8 |
| Packyears** | 24 ± 24 | .097 | 16 ± 29 | n. s. | 9 ± 15 |
| AHI** [n/h] | 14.3 ± 6.8 | < .001 | 48.5 ± 12.3 | < .001 | 2.2 ± 1.2 |
| ODI** [n/h] | 13.0 ± 7.5 | < .001 | 43.1 ± 18.6 | < .001 | 2.6 ± 1.4 |
| Vascular strain analysis of common carotid arteries | | | | | |
| r.Vel** [cm/s] | .076 ± .039 | n. s. | .036 ± .019 | < .01 | .115 ± .079 |
| r-Dis [mm] | .106 ± .053 | < .05 | .044 ± .024 | < .001 | .163 ± .079 |
| r.Str [%] | 2.740 ± 1.078 | < .05 | 1.561 ± .974 | < .001 | 3.783 ± 1.113 |
| c.Str [%] | 2.100 ± 1.003 | < .05 | 1.074 ± .610 | < .001 | 3.694 ± 1.950 |
| r.StrR [1/s] | .222 ± .067 | n. s. | .157 ± .112 | < .05 | .281 ± .115 |
| c.StrR** [1/s] | .148 ± .061 | n. s. | .084 ± .046 | < .01 | .260 ± .185 |
| Vascular strain analysis of brachial arteries | | | | | |
| r.Vel [cm/s] | .026 ± .011 | n. s. | .026 ± .016 | n. s. | .041 ± .021 |
| r-Dis [mm] | .031 ± .017 | .073 | .027 ± .017 | < .05 | .053 ± .035 |
| r.Str [%] | 1.246 ± .512 | < .05 | 1.692 ± 1.032 | n. s. | 2.412 ± 1.239 |
| c.Str [%] | 1.017 ± .520 | n. s. | .934 ± .751 | n. s. | 1.519 ± .554 |
| r.StrR [1/s] | .125 ± .043 | .068 | .180 ± .107 | n. s. | .203 ± .060 |
| c.StrR [1/s] | .088 ± .036 | n. s. | .092 ± .068 | n. s. | .121 ± .042 |
| Vascular strain analysis of femoral arteries | | | | | |
| r.Vel** [cm/s] | .050 ± .026 | n. s. | .040 ± .029 | n. s. | .046 ± .023 |
| r-Dis [mm] | .065 ± .033 | n. s. | .046 ± .031 | n. s. | .060 ± .034 |
| r.Str** [%] | 2.045 ± 1.137 | n. s. | 1.993 ± .904 | n. s. | 2.915 ± 1.795 |
| c.Str [%] | 1.324 ± .569 | n. s. | 1.186 ± .869 | n. s. | 1.237 ± .656 |
| r.StrR** [1/s] | .170 ± .093 | n. s. | .179 ± .094 | n. s. | .237 ± .115 |
| c.StrR [1/s] | .103 ± .044 | n. s. | .089 ± .061 | n. s. | .094 ± .044 |
