# Supplementary material for: Impairment of vascular strain in patients with obstructive sleep apnea
Source: PLoS One. 2018 Feb 28;13(2):e0193397. doi: 10.1371/journal.pone.0193397 (PMC5831412; doi:10.1371/journal.pone.0193397)
Supplement: S2 Table — * vs. control; ** non-Gaussian distributed; HDL high density lipoprotein; LDL low density lipoprotein; Lip(a) lipoprotein a; CRP high sensitive C-reactive protein; IL-6 interleukin-6; IL-2-r interleukin-2-receptor; Fib fibrinogen; Leu leucocytes; Ery erythrocytes; Hb hemoglobin; Hkt hematocrit; Thrombo thrombocytes; Neutro neutrophiles; Lymph lymphocytes; Mono monocytes; Eos eosinophiles; Baso basophiles. (DOCX) [file pone.0193397.s002.docx]

|  | Mild-to-moderate OSA | | Severe OSA | | Control |
| --- | --- | --- | --- | --- | --- |
|  | **Value** | **p*** | **Value** | **p*** | **Value** |
| Total cholesterol [mg/dl] | 200 ± 39 | n. s. | 213 ± 40 | .05 | 186 ± 39 |
| HDL** [mg/dl] | 49 ± 10 | n. s. | 46 ± 15 | n. s. | 48 ± 11 |
| LDL [mg/dl] | 128 ± 32 | n. s. | 141 ± 36 | .06 | 119 ± 32 |
| Lip(a)** [mg/l] | 257 ± 331 | n. s. | 183 ± 235 | n. s. | 156 ± 136 |
| CRP** [mg/l] | 4.3 ± 4.8 | n. s. | 3.7 ± 4.3 | n. s. | 1.8 ± 1.5 |
| IL-6** [pg/ml] | 2.3 ± .9 | n. s. | 3.4 ± 3.4 | n. s. | 2.6 ± .8 |
| IL-2-r [U/ml] | 483 ± 135 | n. s. | 474 ± 181 | n. s. | 489 ± 145 |
| Fib** [g/l] | 3.4 ± .8 | n. s. | 3.2 ± .6 | n. s. | 3.0 ± .5 |
| D-dimer** [mg/l FEU] | .35 ± .16 | n. s. | .31 ± .15 | n. s. | .32 ± .10 |
| Leu [G/l] | 7.22 ± 1.74 | n. s. | 7.21 ± 1.62 | n. s. | 6.51 ± 1.19 |
| Ery [T/l] | 4.79 ± .40 | n. s. | 4.98 ± .52 | n. s. | 4.72 ± .50 |
| Hb [g/dl] | 14.4 ± 1.2 | n. s. | 14.8 ± 1.4 | .05 | 13.8 ± 1.5 |
| Hkt** [%] | 42 ± 3 | n. s. | 43 ± 4 | < .05 | 40 ± 4 |
| Thombo** [G/l] | 250 ± 57 | n. s. | 249 ± 57 | n. s. | 228 ± 45 |
| Neutro [%] | 57.8 ± 8.1 | n. s. | 57.1 ± 7.4 | n. s. | 61.0 ± 8.2 |
| Neutro [G/l] | 4.22 ± 1.29 | n. s. | 4.15 ± 1.30 | n. s. | 3.97 ± .90 |
| Lymph [%] | 30.8 ± 7.9 | n. s. | 30.0 ± 6.9 | n. s. | 28.0 ± 7.9 |
| Lymph [G/l] | 2.20 ± .63 | n. s. | 2.15 ± .56 | n. s. | 1.83 ± .63 |
| Mono [%] | 8.0 ± 1.9 | n. s. | 9.4 ± 2.1 | < .05 | 7.9 ± 2.0 |
| Mono [G/l] | .57 ± .17 | n. s. | .66 ± .15 | < .01 | .51 ± .16 |
| Eos** [%] | 2.7 ± 1.6 | n. s. | 2.6 ± 2.2 | n. s. | 2.4 ± 1.6 |
| Eos** [G/l] | .20 ± .63 | n. s. | .19 ± .17 | n. s. | .16 ± .11 |
| Baso** [%] | .6 ± .3 | n. s. | .8 ± .3 | n. s. | .7 ± .4 |
| Baso** [G/l] | .05 ± .02 | n. s. | .06 ± .03 | n. s. | .05 ± .02 |
