# Supplementary material for: Impairment of vascular strain in patients with obstructive sleep apnea
Source: PLoS One. 2018 Feb 28;13(2):e0193397. doi: 10.1371/journal.pone.0193397 (PMC5831412; doi:10.1371/journal.pone.0193397)
Supplement: S3 Table — * non-Gaussian distributed; n. s. not significant; BMI Body Mass Index; AHI Apnea-Hypopnea-Index; ODI Oxygen-Desaturation-Index; HDL high density lipoprotein; LDL low density lipoprotein; CRP high sensitive C-reactive protein; Mono monocytes; Eos eosinophiles; Baso basophiles; r.Vel radial velocity; r.Dis radial displacement; r.Str radial strain; c.Str circumferential strain; r.StrR radial strain rate; c.StrR circumferential strain rate. (DOCX) [file pone.0193397.s003.docx]

|  | Statin | Non-Statin | p |
| --- | --- | --- | --- |
| Age [Years] | 64 ± 12 | 63 ± 14 | n. s. |
| BMI [kg/m^2^] | 31.3 ± 5.5 | 30.5 ± 5.1 | n. s. |
| Packyears* | 12 ± 16 | 15 ± 18 | n. s. |
| AHI* [n/h] | 28.2 ± 21.9 | 28.3 ± 20.5 | n. s. |
| ODI* [n/h] | 27.4 ± 22.0 | 24.4 ± 20.5 | n. s. |
| Blood testing | | | |
| Total cholesterol [mg/dl] | 171 ± 30 | 210 ± 38 | < .001 |
| HDL [mg/dl] | 46 ± 11 | 48 ± 9 | n. s. |
| LDL [mg/dl] | 107 ± 31 | 139 ± 34 | < .01 |
| CRP* [mg/l] | 3.4 ± 3.5 | 4.6 ± 5.1 | n. s. |
| Mono [G/l] | .73 ± .21 | .63 ± .17 | n. s. |
| Eos* [G/l] | .17 ± .10 | .22 ± .15 | n. s. |
| Baso* [G/l] | .05 ± .03 | .05 ± .02 | n. s. |
| Vascular strain analysis of common carotid arteries | | | |
| r.Vel* [cm/s] | .071 ± .045 | .062 ± .055 | n. s. |
| r.Dis [mm] | .106 ± .073 | .089 ± .071 | n. s. |
| r.Str [%] | 2.780 ± 1.839 | 2.556 ± 1.617 | n. s. |
| c.Str* [%] | 2.498 ± 1.804 | 1.768 ± 1.287 | n. s. |
| r.StrR [1/s] | .227 ± .129 | .205 ± .122 | n. s. |
| c.StrR* [1/s] | .169 ± .110 | .123 ± .095 | n. s. |
